# Unilateral lateral rectus palsy: an unusual presentation of pineal epidermoid cyst

**DOI:** 10.11604/pamj.2020.36.252.24378

**Published:** 2020-08-06

**Authors:** Koushik Handattu, Ramesh Bhat Yellanthoor, Sandesh Kini

**Affiliations:** 1 Department of Paediatrics, Kasturba Medical College, MAHE University, Manipal, India

**Keywords:** Pineal gland cyst, sixth nerve palsy, child

## Abstract

An 11-year-old boy presenting with sudden onset double vision, headache and neck pain was found to have left lateral rectus palsy, papilledema and neck rigidity. An initial diagnosis of benign intracranial hypertension was considered. However, magnetic resonance imaging (MRI) of the brain surprisingly discovered the pineal gland cyst. He underwent Krause´s procedure for the excision biopsy of the cyst. Histopathological examination confirmed epidermoid cyst. Postoperatively, he had transient ataxia and upgaze palsy but recovered well. He was asymptomatic during the first and third-month follow-ups. The case highlights the unusual presentation of a rare intracranial tumor, pineal epidermoid cyst. Neuroimaging and timely surgery lead to a good outcome.

## Introduction

Epidermoid cysts are a group of slow growing intracranial tumors representing approximately 1% of all central nervous system tumors. Furthermore, pineal location of epidermoid cyst is extremely rare [1,2]. Headache and ataxia are the common modes of presentation [2]. We report an unusual presentation of this rare tumor, diplopia and unilateral lateral rectus palsy, in an adolescent boy.

## Patient and observation

An eleven year old boy from rural South India was referred to our centre by an ophthalmologist for sudden onset diplopia. The diplopia was particularly complained when looking towards the left side. He also complained blurring of vision and frequent eye blinking in both eyes. There was no headache, vomiting, speech disturbance, seizures or weakness of limbs. There was history of trivial trauma to head while playing in the school, three weeks ago. There was no external injury. He developed some restriction of neck movements following the incident which improved subsequently. His birth history and developmental history were unremarkable. His immunization was appropriate for age. On examination, he weighed 32.6 kilograms. His height was 139cm, head circumference, 52cm and body mass index was 17.1kg/m^2^. His vital signs were normal. Central nervous system examination revealed normal higher mental functions, grade 3 papilledema bilaterally, left lateral rectus palsy, nystagmus on left lateral gaze and neck stiffness. Other cranial nerves, motor and sensory system were normal. There were no cerebellar signs and the gait was normal. He was admitted in the intensive care unit for further evaluation and monitoring. An initial diagnosis of benign intracranial hypertension was considered and he was started on anti- cerebral edema measures pending neuroimaging.

**Figure 1:**
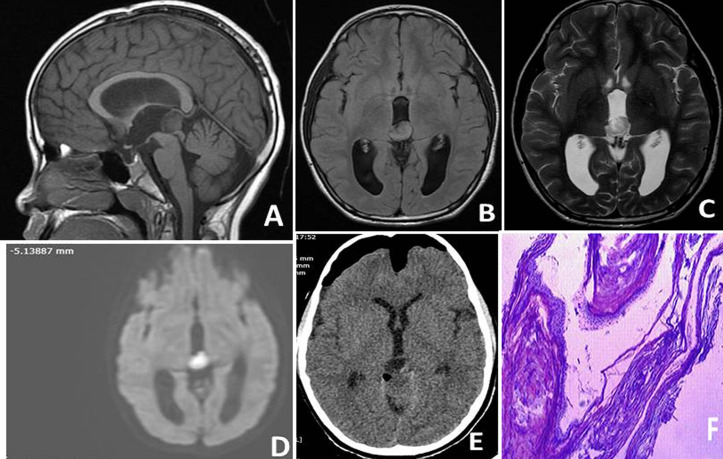
A,B) magnetic resonance imaging (MRI) of the brain shows a well-defined lesion measuring 1.6cm x 1.5cm x 1.6cm in the pineal region. The lesion was hyper intense on T1 and T2 weighted images; C) the lesion shows no post-contrast enhancement; D) the lesion shows intense diffusion restriction; E) the postoperative MRI shows complete removal of cyst; F) histopathology (haematoxylin & eosin stain) shows cyst wall lined by stratified squamous epithelium with abundant luminal lamellated keratin consistent with epidermoid cyst

His complete blood count, electrolytes, renal and hepatic function tests were normal. Magnetic resonance imaging of the brain revealed a well-defined lesion measuring 1.6cm x 1.5cm x 1.6cm in the pineal region. The pineal gland was not visualized separately. The lesion was hyper intense on T1 and T2 weighted images (Figure 1A and Figure 1B) with no post-contrast enhancement (Figure 1C). It also showed intense diffusion restriction (Figure 1D). The lesion was causing compression of superior colliculus on its antero-inferior aspect and posteromedial portions of both thalami on either side with obstruction to the third ventricular outflow leading to mild obstructive hydrocephalus. Radiologists opined the possibilities of pineal cyst with hemorrhage or pineal epidermoid cyst. He underwent excision biopsy through Krause´s approach. The postoperative MRI showed complete removal of cyst (Figure 1E). Histopathology examination confirmed pineal epidermoid cyst (Figure 1F). In the postoperative period, he had ataxia and upgaze palsy, which resolved in due course. The left lateral rectus palsy had improved. He recovered completely and discharged from the hospital on post-operative day 7 without any neurological deficits. Follow up at the end of one month and three months, he was symptom free and his neurological examination was normal.

## Discussion

Epidermoid cysts represent misplaced ectodermal cells during division of neurectoderm around third to fourth week of life. Epidermoid cysts are rare forms of intracranial tumors. These cysts grow slowly, but attain relatively large size and have a tendency to invade the local tissues [3]. The common intracranial location is the cisterns around the tentorial hiatus. The pineal location of epidermoid cyst is much rare. Pineal epidermoid cysts usually have no sex predilection. They commonly present in third decade of life. They present early compared to epidermoid cysts elsewhere in brain because of close proximity to aqueduct of Sylvius. Symptoms are often subtle, often resulting from neuronal distortion and stretch. They include headache, ataxia, hydrocephalus with features of raised intracranial tension [4]. In the present case child presented with headache, neck pain, left lateral rectus palsy and papilledema. This presentation has led us to think of benign intracranial hypertension at first and the history of trivial trauma preceding the onset of illness further confused the diagnosis until the magnetic resonance imaging (MRI) scan of brain.

MRI is the investigation of choice for evaluation of pineal gland tumors, which can differentiate epidermoid cyst from other intra axial tumors. MRI of cyst shows hyperintensities on T1 and T2 with no post contrast enhancement with intense diffusion restriction [4]. These features were observed in the present case. Complete surgical excision is the treatment of choice, limited by challenging location of tumor. Many surgical approaches are tried including infratentorial supracerebellar approach, occipital transtentorial approach, interhemispheric transcallosal approach and transventricular approach [3,5]. Irrespective of approach all authors cautioned about excessive dissection of capsule from adjoining veins. In this case child was operated with infratentorial supracerebellar (Krause´s) approach. The ataxia and upward gaze palsy observed on the first postoperative day gradually subsided by the end of first post-operative week. This transient phenomenon is probably secondary to quadrigeminal plate injury. Child recovered well in due course and found to be asymptomatic with no other neurodeficits by first month and third month follow up.

## Conclusion

Intracranial epidermoid cysts are quite rare and grow slowly. They usually present with features of raised intracranial pressure. The diplopia and unilateral lateral rectus palsy, the unique presentation in the present case, initially led us to think of benign intracranial hypertension. However, MRI brain scan detected the exact location of the pineal cyst. Though challenging, surgical resection of the cyst by an expert could lead to fairly good results.
